# Harassment of health professionals by the infant food industry at scientific events

**DOI:** 10.11606/s1518-8787.2022056003398

**Published:** 2022-07-21

**Authors:** Ana Carla da Cunha Ferreira Velasco, Maria Inês Couto de Oliveira, Cristiano Siqueira Boccolini

**Affiliations:** I Universidade Federal Fluminense Instituto de Saúde Coletiva Programa de Pós-Graduação em Saúde Coletiva Niterói RJ Brasil Universidade Federal Fluminense . Instituto de Saúde Coletiva . Programa de Pós-Graduação em Saúde Coletiva . Niterói , RJ , Brasil; II Universidade Federal Fluminense Instituto de Saúde Coletiva Departamento de Epidemiologia e Bioestatística Niterói RJ Brasil Universidade Federal Fluminense . Instituto de Saúde Coletiva . Departamento de Epidemiologia e Bioestatística . Niterói , RJ , Brasil; III Fundação Oswaldo Cruz Instituto de Comunicação e Informação Científica e Tecnológica em Saúde Laboratório de Informação em Saúde Rio de Janeiro RJ Brasil Fundação Oswaldo Cruz . Instituto de Comunicação e Informação Científica e Tecnológica em Saúde . Laboratório de Informação em Saúde . Rio de Janeiro , RJ , Brasil

**Keywords:** Breast-Milk Substitutes, Legislation, Food, Food Publicity, Scientific and Educational Events, Health Personnel

## Abstract

**OBJECTIVE:**

To analyze the receipt of sponsorships from breast-milk substitute companies by health professionals in scientific events.

**METHODS:**

Multicenter study (Multi-NBCAL) performed from November 2018 to November 2019 in six cities in different Brazilian regions. In 26 public and private hospitals, pediatricians, nutritionists, speech therapists, and a hospital manager were interviewed using a structured questionnaire. Descriptive analyses were carried out regarding the health professionals’ knowledge about the *Norma Brasileira de Comercialização de Alimentos para Lactentes e Crianças de Primeira Infância, Bicos, Chupetas e Mamadeiras* (NBCAL – Brazilian Code of Marketing of Infant and Toddlers Food and Childcare-related Products), companies sponsoring scientific events, and material or financial sponsorships received, according to profession.

**RESULTS:**

We interviewed 217 health professionals, mainly pediatricians (48.8%). Slightly more than half of the professionals (54.4%) knew NBCAL, most from Baby-friendly Hospitals. Most health professionals (85.7%) attended scientific events in the last two years, more than half of them (54.3%) sponsored by breast-milk substitute companies, especially Nestlé (85.1%) and Danone (65.3%). These professionals received sponsorships in the events, such as office supplies (49.5%), meals or invitations to parties (29.9%), promotional gifts (21.6%), payment of the conference registration fee (6.2%) or ticket to the conference (2.1%).

**CONCLUSION:**

The infant food industries violate NBCAL by harassing health professionals in scientific conferences, offering diverse material and financial sponsorships.

## INTRODUCTION

The first years of a child’s life are characterized by the speed of growth and development, and breast milk is recognized as the gold standard for infant nutrition, as it presents modulatory function ^1^ , reduces infant morbidity and mortality, and provides immune protection to the child ^2^ . The World Health Organization (WHO) recommends exclusive breastfeeding during the first six months of life and breastfeeding complemented by healthy foods up to two years of age or more ^3^ .

Aiming to protect breastfeeding, the WHO and the United Nations Children’s Fund (UNICEF), in the Joint Meeting on Infant and Young Child Feeding in 1979, proposed the creation of a set of regulatory standards that originated, in 1981, the International Code of Marketing of Breast-milk Substitutes ^4^ .

Inspired by the Code, the first Brazilian legislation to regulate the advertising and marketing of breast-milk substitutes was created in 1988. It underwent several updates over the years and is currently called *Norma Brasileira de Comercialização de Alimentos para Lactentes e Crianças de Primeira Infância, Bicos, Chupetas e Mamadeiras* (NBCAL – Brazilian Code of Marketing of Infant and Toddlers Food and Childcare-related Products), acquiring force of law in 2006 ^5^ . Law No. 11.265/2006 establishes that manufacturers of infant formulas, toddlers formulas, milks, commercial complementary foods, and related childcare products, as well as distributors of these products, can not offer material or financial sponsorship to individuals. Such sponsorship is only allowed to scientific education and research institutions, or to nationally recognized associations of pediatricians and nutritionists ^6^ . Decree No. 9.579/2018, which regulates this Law, defines sponsorship as the funding of materials, research, events, or of the participation of health professionals in activities, or any kind of incentive. Companies are allowed to provide one sample of infant formula or baby food to pediatricians or nutritionists at the time of the product launch ^7^ . The *Agência Nacional de Vigilância Sanitária* (ANVISA – Brazilian Health Regulatory Agency) is responsible for monitoring and punishing companies that violate NBCAL ^8^ .

Although the government and civil society organizations invest in protecting breastfeeding, marketing strategies of multinational corporations that produce breast-milk substitutes are constantly renewed and proved to be effective in increasing sales, which shows the importance of comprehensive national laws and proper monitoring of their practices ^9^ . As marketing strategies, companies that produce infant formulas, teats, pacifiers, and baby bottles have been presenting their products as equally good or superior to breastfeeding ^9^ and using the influence of health professionals on mothers and families to increase their market ^10^ .

The infant food industry attends scientific events to present its products and influence health professionals. The purpose of this industry’s harassment is to create links with health professionals, teachers, and students ^11^ . This study aimed to analyze the receipt of sponsorships from breast-milk substitute companies by health professionals in scientific events.

## METHODS

This is a cross-sectional study, part of the *Estudo Multicêntrico de Avaliação do Cumprimento da NBCAL* (Multi-NBCAL – Multicenter Study for NBCAL Compliance Assessment). The project was approved by the Fiocruz research ethics committee under protocol no. 2.912.729/2018.

The survey team consisted of a coordinator and vice coordinator specialist in surveys and NBCAL, as well as a field supervisor and from one to four interviewers per city. All supervisors and interviewers participated in 20-hour theoretical and practical courses on NBCAL—with practical sessions to assess its compliance in hospitals, drugstores, and supermarkets—and were instrumentalized in online data collection.

This study was carried out in hospitals with obstetric beds, according to a protocol adapted from the NetCode (NetCode periodic assessment) ^12^ , which recommends the random selection of 10 maternity hospitals in the capital or the largest city of the assessed country, as well as the selection of more maternity hospitals in other settings, if financially possible.

The survey coordination chose to compare different types of hospitals, located in capitals with different Municipal Human Development Indexes and in a city in the interior of Brazil, in order to decentralize the study from the capital or the biggest city due to the great diversity and large extension of the country. The cities were chosen intentionally, according to the availability of institutions and researchers focused on the subject ^13^ . The survey was performed from November 2018 to November 2019 in six cities in different Brazilian regions: Rio de Janeiro, RJ (six hospitals), São Paulo, SP (five hospitals), Ouro Preto, MG (one hospital), Florianópolis, SC (four hospitals), João Pessoa, PB (six hospitals), and Brasília, DF (four hospitals).

In each city, all hospitals with obstetric beds with more than 500 births per year were listed via consultation in the *Cadastro Nacional de Estabelecimentos de Saúde* (CNES – National Registry of Health Establishments) ^a^ . They were categorized as public hospitals accredited by the Baby-friendly Hospital Initiative (BFHI), public hospitals non-accredited by the BFHI, and private hospitals. This study did not include private Baby-friendly Hospitals because, at the time, Brazil only had nine hospitals of this type, all located in interior cities ^b^ . The sample would come from two hospitals per stratum in each city and, if the number were higher than this amount in the municipality, they would be randomly selected. However, only four hospitals had obstetric beds in Florianópolis, SC, and all of the public ones were Baby-friendly Hospitals. In Ouro Preto, MG, only one hospital had obstetric beds and it was also a Baby-friendly Hospital. In João Pessoa, PB, all public hospitals were accredited by this initiative. In two cities, three maternity hospitals refused to participate in the study. Difficulties in obtaining the *Termos de Autorização Institucional* (TAI – Institutional Authorization Agreement) prevent this study from reaching the previously established sample ^13^ . Finally, this study assessed 11 Baby-friendly Hospitals, six non-accredited public hospitals, and nine private hospitals.

The interviewers carried out the survey in a single day, personally interviewing in each maternity a hospital manager, five pediatricians, and five nutritionists and/or speech therapists—a fixed number of professionals per maternity, according to the NetCode monitoring protocol ^12^ . If less than 10 professionals of these categories were working in the maternity hospital on the day of the survey, all of them were interviewed. If more than 10 professionals were working, a hospital manager listed the pediatricians, nutritionists, and speech therapists present on the day of the survey and up to five pediatricians, five nutritionists and/or speech therapists, and one hospital manager were randomly selected. All interviewees signed an informed consent form ^13^ .

For the interviews, an online electronic questionnaire was applied using the MAGPI application, installed on mobile phones with Android operating system, with intuitive interface for data entry, electronic hosting on Fiocruz servers, data collection online monitoring, and possibility of exporting data to the most common statistical packages ^14^ .

The questionnaire used was adapted from the International Baby Food Action Network (IBFAN) ^c^ and NetCode ^12^ monitoring questionnaire, with content validation by expert panel ^13^ . The interviewees were asked about: (1) health professional profile: sex, age, skin color, profession, time since graduation, and sectors of activity (more than one sector could be mentioned: rooming-in, neonatal ICU, human milk bank); (2) knowledge about NBCAL: yes, regular (corresponding to the answers “I have an idea” and “I have heard about it”), or no; mentioned at least one product covered by NBCAL: yes (infant formula, teat, baby bottle, pacifier, toddlers formula, milk, or commercial complementary food) or no; participation in training in NBCAL by a course or class: yes or no; agency responsible for training: government agency, professional association, industry, private (corresponding to private university, private hospital, paid course, or other company, as distributors); participation in training in infant feeding: yes or no; (3) participation in a conference or symposium on infant feeding in the last two years: yes or no; event sponsorship by infant food or related products companies: yes or no; sponsor company (more than one company could be mentioned: Nestlé, Danone, Abbott, Mead Johnson, other); sponsorship received by the health professional (more than one could be mentioned: payment of the conference registration fee, ticket to the conference, meal or invitations to parties, office supplies, promotional gift; receipt of free samples of breast-milk substitutes): yes or no.

Descriptive analyses were performed according to the type of hospital, regarding the distribution of health professionals interviewed by city, their profile, their knowledge about NBCAL, their training in NBCAL, and the agency responsible for their course and training in infant feeding. The proportion of each agency responsible for training in NBCAL was estimated only among professionals who were trained.

To verify the homogeneity of the health professionals’ characteristics, as well as their knowledge and training in NBCAL and infant feeding, according to the type of hospital, a Pearson’s chi-squared test was applied, considering a 5% level of statistical significance.

The proportion of health professionals who attended scientific events in the last two years, according to profession, was analyzed. The proportion of those who reported that the event was sponsored by breast-milk substitute companies was estimated and each professional presented the companies that sponsored the events, being able to mention more than one company.

Finally, among the health professionals who attended scientific events sponsored by this industry, the frequency of those who received material or financial sponsorships in these events, as well as the frequency of those who received free samples of breast-milk substitutes, according to profession, was analyzed.

## RESULTS

We interviewed 217 health professionals who worked in 26 hospitals in six Brazilian cities. Of these professionals, 44.2% worked in public hospitals accredited by the BFHI, 26.3% in public hospitals non-accredited by the BFHI, and 29.5% in private hospitals. Most health professionals interviewed were from Rio de Janeiro, São Paulo, and João Pessoa ( Table 1 ). Four health professionals refused to participate in the study (1.8%).

**Table 1:** Health professionals interviewed by municipality, according to the type of hospital. Multi-NBCAL, 2018–2019.

Municipality	Public Baby-friendly Hospitals		Non-accredited public hospitals		Private hospitals		Total
	n	%	n	%	n	%	n
Rio de Janeiro	25	37.3	31	46.3	11	16.4	67
São Paulo	9	22.5	19	47.5	12	30	40
Ouro Preto	6	100	0	0	0	0	6
Florianópolis	18	56.3	0	0	14	43.8	32
João Pessoa	30	66.7	0	0	15	33.3	45
Brasília	8	29.6	7	25.9	12	44.4	27
Total	96	44.2	57	26.3	64	29.5	217

NBCAL: *Norma Brasileira de Comercialização de Alimentos para Lactentes e Crianças de Primeira Infância, Bicos, Chupetas e Mamadeiras* .

Most professionals interviewed were women, especially in private hospitals. Most health professionals were white, except in private hospitals, and 12.4% of the professionals did not declare their skin color. Among the interviewees, 48.8% were pediatricians, 29% were nutritionists, 18% were speech therapists, and 4.1% were hospital managers, and this distribution was relatively homogeneous among the different types of hospitals. Most professionals were 35 to 59 years old and had graduated from 11 to 24 years ago. The most frequent sectors of activity were neonatal ICU and rooming-in ( Table 2 ).

**Table 2:** Characteristics of the health professionals interviewed, according to the type of hospital. Multi-NBCAL, 2018–2019.

Characteristics	Public Baby-friendly Hospitals		Non-accredited public hospitals		Private hospitals		Total		p
	n	%	n	%	n	%	n	%	
Sex									
Men	17	17.7	8	14	2	3.1	27	12.4	0.022
Women	79	82.3	49	86	62	96.9	190	87.6	
Age									
20–34	24	25	13	22.8	24	37.5	61	28.1	0.247
35–59	65	67.7	39	68.4	33	51.6	137	63.1	
≥ 60	7	7.3	5	8.8	7	10.9	19	8.8	
Skin color									
White	63	65.6	37	64.9	25	39.1	125	57.6	0.001
Black	5	5.2	2	3.5	9	14.1	16	7.4	
Mixed-race	23	24	6	10.5	18	28.1	47	21.7	
Asian	1	1	1	1.8	0	0	2	0.9	
Undeclared	4	4.2	11	19.3	12	18.8	27	12.4	
Profession									
Pediatrician	45	46.9	32	56.1	29	45.3	106	48.8	0.184
Nutritionist	25	26	19	33.3	19	29.7	63	29	
Speech therapist	19	19.8	6	10.5	14	21.9	39	18	
Other	7	7.3	0	0	2	3.1	9	4.1	
Time since graduation									
≤ 10 years	29	30.2	17	29.8	27	42.2	73	33.6	0.250
11–24 years	41	42.7	19	33.3	23	35.9	83	38.2	
≥ 25 years	26	27.1	21	36.8	14	21.9	61	28.1	
Sectors of activity									
Rooming-in	50	52.1	29	50.9	32	50	111	51.2	0.966
Neonatal ICU	57	59.4	34	59.6	46	71.9	137	63.1	0.225
Human milk bank	7	7.3	6	10.5	5	7.8	18	8.3	0.771
Total	96	100	57	100	64	100	217	100	

NBCAL: *Norma Brasileira de Comercialização de Alimentos para Lactentes e Crianças de Primeira Infância, Bicos, Chupetas e Mamadeiras* ; ICU: intensive care unit.

A total of 54.4% of health professionals knew NBCAL and 59.9% were able to mention at least one product covered by this legislation. This knowledge was higher among professionals who worked in Baby-friendly Hospitals and lower among those who worked in non-accredited public hospitals. A total of 30% of the interviewees participated in courses or classes on NBCAL, and this number was higher among professionals who worked in Baby-friendly Hospitals (40.6%). Among those trained in NBCAL, 93.8% reported that it was performed by a government agency. The participation in courses or classes on infant feeding was high (93.5%) in all types of hospitals ( Table 3 ).

**Table 3:** Health professionals’ knowledge about NBCAL and training in NBCAL and infant feeding, according to the type of hospital. Multi-NBCAL, 2018–2019.

Knowledge/training	Public baby-friendly hospitals		Non-accredited public hospitals		Private hospitals		Total		p
	n	%	n	%	n	%	n	%	
Knew NBCAL									
Yes	65	67.7	23	40.4	30	46.9	118	54.4	0.001
Regular ^a^	14	14.6	6	10.5	13	20.3	33	15.2	
No	17	17.7	28	49.1	21	32.8	66	30.4	
Mentioned NBCAL products									
Yes	73	76	25	43.9	32	50	130	59.9	< 0.001
No	23	24	32	56.1	32	50	87	40.1	
Course/class on NBCAL									
Yes	39	40.6	14	24.6	12	18.8	65	30	0.007
No	57	59.4	43	75.4	52	81.3	152	70	
Course responsible									
Government agencies	39	100	13	92.9	9	75	61	93.8	0.001
Professional association	0	0	0	0	0	0	0	0	0
Industry	0	0	0	0	0	0	0	0	0
Private ^b^	0	0	1	7.1	2	16.7	3	4.6	0.243
Other	0	0	0	0	1	8.3	1	1.5	0.301
Course/class on infant feeding									
Yes	92	95.8	50	87.7	61	95.3	203	93.5	0.113
No	4	4.2	7	12.3	3	4.7	14	6.5	
Total	96	100	57	100	64	100	217	100	

NBCAL: *Norma Brasileira de Comercialização de Alimentos para Lactentes e Crianças de Primeira Infância, Bicos, Chupetas e Mamadeiras* .

^a^ Regular corresponds to the answers: “I have an idea” or “I have heard about it”.

^b^ Private corresponds to the answers: university, private hospital, or paid course.

Most health professionals attended scientific events in the last two years (85.7%) and 54.3% of them reported that breast-milk substitute companies sponsored these events, mainly pediatricians (71.1%). Nestlé (85.1%) and Danone (65.3%) were the most mentioned sponsor companies of scientific events ( Table 4 ).

**Table 4:** Participation in scientific events, infant food industry sponsorship, and sponsor companies, according to profession. Multi-NBCAL, 2018–2019.

Participation/sponsorship	Pediatrician		Nutritionist		Speech therapist		Other		Total	
	n	%	n	%	n	%	n	%	n	%
Participation in congresses										
Yes	97	91.5	45	71.4	37	94.9	7	77.8	186	85.7
No	9	8.5	18	28.6	2	5.1	2	22.2	31	14.3
Event sponsored by the infant food industry ^a^										
Yes	69	71.1	18	40	13	35.1	1	14.3	101	54.3
No	28	28.9	27	60	24	64.9	6	85.7	85	45.7
Sponsor company ^b^										
Nestlé	60	87	14	77.8	11	84.6	1	100	86	85.1
Danone	50	72.5	9	50	6	46.2	1	100	66	65.3
Abbott	27	39.1	3	16.7	0	0	1	100	31	30.7
Mead Johnson	27	39.1	3	16.7	0	0	0	0	30	29.7
Other	3	4.3	2	11.1	0	0	0	0	5	5

NBCAL: *Norma Brasileira de Comercialização de Alimentos para Lactentes e Crianças de Primeira Infância, Bicos, Chupetas e Mamadeiras* .

^a^ Estimated among professionals who attended conferences in the last two years.

^b^ Each professional could mention more than one sponsor company.

Most health professionals who attended scientific events sponsored by breast-milk substitute companies received material or financial sponsorship from the sponsor company, such as office supplies (49.5%), meals or invitations to parties (29.9%), promotional gifts (21.6%), payment of the conference registration fee (6.2%), or ticket to the conference (2.1%). About one third of the interviewees did not receive sponsorship. A total of 22.7% of health professionals received free samples of breast-milk substitutes: 25% of pediatricians, 18.8% of nutritionists, a speech therapist, and another health professional ( Table 5 ).

**Table 5:** Receipt of sponsorships and free samples in scientific conferences, according to profession. Multi-NBCAL, 2018–2019.

Sponsorship	Pediatrician		Nutritionist		Speech therapist		Other		Total	
	n	% ^a^	n	%	n	%	n	%	n	%
Material or financial sponsorship in the conference										
Office supplies	30	44.1	10	62.5	8	66.7	0	0	48	49.5
Meal or party	21	30.9	6	37.5	2	16.7	0	0	29	29.9
Promotional gift	15	22.1	5	31.2	1	8.3	0	0	21	21.6
Conference registration	3	4.4	2	12.5	1	8.3	0	0	6	6.2
Ticket	2	2.9	0	0	0	0	0	0	2	2.1
No sponsorship received	27	39.7	2	12.5	4	33.3	1	100	34	35.1
Sample in the conference										
Free sample	17	25	3	18.8	1	8.3	1	100	22	22.7
Total	68	100	16	100	12	100	1	100	97	100

NBCAL: *Norma Brasileira de Comercialização de Alimentos para Lactentes e Crianças de Primeira Infância, Bicos, Chupetas e Mamadeiras* .

^a^ Percentages estimated from the total number of professionals who attended events sponsored by the infant food industry (four interviewees did not answer).

## DISCUSSION

Although prohibited by Law No. 11.265/2006, almost two thirds of the professionals who attended scientific events sponsored by breast-milk substitute companies received material or financial sponsorship in these events. Breast-milk substitute companies harass pediatricians, nutritionists, and speech therapists by paying registration fees and tickets to conferences—both high-cost items—as well as by inviting to parties and meals and offering bags, pens, notebooks, notepads, calendars, and promotional gifts—all low-cost items. Besides harassing health professionals by financial sponsorship and gifts, in scientific events, breast-milk substitute companies disseminate information favorable to the use of their products ^15^ , aiming to influence pediatricians, whose relationship with patients is considered “fiduciary” in the prescription of these products, as they have specialized knowledge, experience, and the confidence of all ^16^ . Companies also establish relationships with opinion leaders and health organizations in order to influence health programs by accessing and placing actors in government policy contexts ^15^ . They also aim to impair regulatory measures, prevent the approval of legislation, delay their implementation, or reverse measures already implemented ^17^ .

The promotion of breast-milk substitutes by its manufacturers and distributors is a barrier to increase breastfeeding worldwide ^18 , 19^ , especially in low- and middle-income countries, where sales of breast-milk substitutes grow 10% per year ^20^ . In these countries, only 37% of children under six months of age are exclusively breastfed, and breastfeeding could save up to 823,000 children every year ^2^ .

NBCAL prohibits breast-milk substitute companies to sponsor individuals, in order to protect mothers from inadequate commercial pressures, since the infant food industry uses various strategies to gain mothers’ loyalty in the consumption of their products ^21^ .

However, in our study, about 60% of pediatricians who attended scientific events sponsored by these companies received material or financial sponsorships in these events. More than 40% received office supplies and almost a third were invited to parties or meals. Nutritionists and speech therapists were also heavily harassed by the infant food industry. A study carried out in Indonesia showed that multinational infant food companies also offers health professionals magnets, posters, thermometers, calendars, growth charts, and frequent visits to them ^22^ . When health professionals accept those gifts, they are indirectly promoting breast-milk substitute companies instead of supporting breastfeeding. Moreover, when health professionals have promotional gifts with the logo of infant food companies, their patients can understand it as an incentive to the use of these products ^23^ .

Grummer-Strawn et al. ^24^ , using data collected on the Internet, observed a strong presence of the infant food industry among pediatricians, as 60% of pediatric associations worldwide received some kind of financial sponsorship from breast-milk substitute companies, especially in the Americas (82%), Europe (66%), and Asia (50%). This reality is similar in Brazil, where a partnership between the *Sociedade Brasileira de Pediatria* (SBP – Brazilian Pediatric Association) and a breast-milk substitute company resulted in the *Programa Jovens Pediatras: Nestlé Nutrition* (Young Pediatricians Program: Nestlé Nutrition), a program to influence the professional training of residents in pediatrics ^d^ .

When a health or nutrition conference in sponsored by an infant food company, it works as an endorsement to that brand. When an event is sponsored by breast-milk substitute companies, the event promoter must then support the use of the industry’s products or practices ^10^ . Financial support or any kind of sponsorship by manufacturers of breast-milk substitutes can build the loyalty of health professionals and professional associations to infant food companies ^22^ . Rea and Toma ^25^ , in one of the first national studies on the subject, highlight the conflicts of interest involved in the health professional–industry relationship, presenting results from a NBCAL monitoring carried out in more than 30 Brazilian cities, which observed ways breast-milk substitute companies use to attract health professionals, such as granting plane tickets, airport pickup, and conference registration.

Among multinational breast-milk substitute companies, Nestlé was the most mentioned by health professionals as sponsor of scientific events. Brazil is the second largest market and the fifth largest country in revenue of Nestlé. This company is the food industry leader, with 15 branches throughout Brazil, besides five distribution centers, four warehouses, and 26 factories ^26^ . Similarly, in Mexico, managers of public and private hospitals pointed Nestlé as the manufacturer of infant food that most contacts them ^27^ .

Although more than half of health professionals knew NBCAL and almost 60% were able to mention at least one product covered by it, less than one third participated in some course or class on the subject. Most courses or classes on NBCAL were performed by government agencies. Professional associations must present a greater commitment in training professionals to comply with NBCAL and the International Code of Marketing of Breast-milk Substitutes, as the lack of training promoted by them is impressive. Understanding this important legislation to protect breastfeeding would not prevent health professionals from promoting breast-milk substitute companies, but it could increase their perception on the conflicts of interest involved and reduce violations of this Law, contributing to the promotion of community health ^28^ .

Current medical school curricula are more focused on teaching their students how to feed babies with infant formula than on supporting mothers to breastfeed with counseling skills ^29^ . A study performed with students from the *Faculdade de Medicina da Universidade Federal de Alagoas* observed that students in the last period know little about the International Code and NBCAL, showing that much must be done to promote teaching about breastfeeding in the medical school ^30^ .

In our study, professionals who worked in Baby-friendly Hospitals presented more knowledge and training in NBCAL, however, the expected was that all professionals knew this legislation, a mandatory content in their education. Continuing education is necessary for professionals with long time since training to improve their knowledge and for systematical training of professionals hired after the hospital being accredited by the BFHI ^31^ . The BFHI was created to provide greater support to the practice of breastfeeding in hospitals with obstetric beds by training health professionals and adjusting hospital routines. A criterion to meet the first of the Ten Steps to Successful Breastfeeding ^24^ , an initiative considered an important strategy, is the compliance with the International Code of Marketing of Breast-milk Substitutes. This strategy affects positively breastfeeding rates and is included in the Sustainable Development Agenda of the United Nations ^31 , 32^ . The sustainability of its actions is a target of continuous monitoring in Brazil ^33^ .

Our study presented limitations: we do not know if the samples received by the pediatricians and nutritionists interviewed corresponded to products being launched, therefore, we could not evaluate to what extent the industry was violating Law No. 11.265/2006. However, in this study, other health professionals also received samples of breast-milk substitutes, violating NBCAL ^7^ . Moreover, we asked health professionals information related to the receipt of sponsorships in conferences in the last two years, subject to memory bias, since forgetting the receipt of sponsorships may be more frequent than reporting non-existent receipt of sponsorships. This bias may underestimate sponsorships received, especially lower-cost gifts.

The NetCode ^12^ methodology proposes to identify the most frequent violations of the International Code ^4^ (and NBCAL ^6^ ) by its method of random selection of maternity hospitals, and interviews. Multi-NBCAL adapted this protocol, assessing 26 maternity hospitals from different cities, including the capital and the largest city of the country. Our limitation was its non-probabilistic sample, which reduces the possibility of generalization of the results. Thus, the findings of this study present the reality of the professionals interviewed in the included maternity hospitals and may not be considered as the reality of the cities assessed. However, the results of this multicenter study are sufficient to discuss potential conflicts of interest in professional practices facing harassment of breast-milk substitutes companies.

A reliability study of the questionnaire is necessary to assess its psychometric properties, however, the constant monitoring of application of most questionnaires by experienced researchers, associated with the rigorous training of interviewers, may have reduced a potential interobserver variability. A reliability analysis of the questionnaire found a Cronbach’s alpha of 0.794 for the questions evaluated in this study (non-tabulated data), a pattern considered high.

Infant food industry marketing strategies focused on health professionals are frequent in education spaces, such as conferences and scientific symposia, showing that the existence of a regulation, even as a law, is not enough to prevent illegal practices to promote breast-milk substitutes, although the effect on child health is widely known ^34^ . Inadequate monitoring of scientific events allows multinational breast-milk substitute companies to improperly involve health professionals in the promotion of their products without any punishment ^19^ .

We recommend the development of government actions to increase knowledge and commitment to current legislation both by companies and health professionals. Moreover, the theme of conflicts of interest must be addressed since undergraduate health courses. As pediatricians are the health professionals with more influence on mothers—as they not only recommend, but also prescribe infant formulas—pediatric associations must collaborate with the continuing education of their professionals with an ethical commitment to breastfeeding and child health. Health surveillance also must have an effective performance in the NBCAL monitoring for a better compliance with current legislation. Conflict of interest cases between the infant food industry and health professionals must be urgently prevented for the protection of breastfeeding and child health.

## Footnotes

a Ministério da Saúde (BR), DATASUS. Cadastro Nacional de Estabelecimentos de Saúde – CNES. Brasília, DF; s.d. [cited 2022 May 22]. Available from: https://cnes.datasus.gov.br/

b Ministério da Saúde. Portal Saúde da Criança NET. IHAC - Iniciativa Hospital Amigo da Criança [cited 2022 May 22]. Available from: http://sisac.datasus.gov.br/saudedacrianca/ihacSobre.html#

c Rede Internacional em Defesa do Direito de Alimentar. Belo Horizonte, MG: IBFAN Brasil; 2022 [cited 2022 May 22]. Available from: http://www.ibfan.org.br

d Aliança pela Alimentação Adequada e Saudável. Porto Alegre, RS; c2020 [cited 2020 Jul 10]. Available from: https://alimentacaosaudavel.org.br/blog/organizacoes-denunciam-conflito-de-interesse-em-curso-da-nestle-para-medicos-residentes-de-pediatria/8180/
