# Supplementary material for: Harassment of health professionals by the infant food industry at scientific events
Source: Rev Saude Publica. 2022 Jul 21;56:70. doi: 10.11606/s1518-8787.2022056003398 (PMC9337847; doi:10.11606/s1518-8787.2022056003398)
Supplement: Translation

# Translation: Assédio da indústria de alimentos infantis a profissionais de saúde em eventos científicos

**Authors:** Ana Carla da Cunha Ferreira Velasco, Maria Inês Couto de Oliveira, Cristiano Siqueira Boccolini

**Affiliations:** I Brasil Universidade Federal Fluminense. Instituto de Saúde Coletiva. Programa de Pós-Graduação em Saúde Coletiva. Niterói, RJ, Brasil; II Brasil Universidade Federal Fluminense. Instituto de Saúde Coletiva. Departamento de Epidemiologia e Bioestatística. Niterói, RJ, Brasil; III Brasil Fundação Oswaldo Cruz. Instituto de Comunicação e Informação Científica e Tecnológica em Saúde. Laboratório de Informação em Saúde. Rio de Janeiro, RJ, Brasil

**Keywords:** Substitutos do Leite Humano, Publicidade de Alimentos, Legislação sobre Alimentos, Eventos Científicos e de Divulgação, Pessoal de Saúde

## Abstract

**OBJETIVO:**

Analisar o recebimento de patrocínios da indústria de substitutos do leite materno por profissionais de saúde em eventos científicos.

**MÉTODOS:**

Inquérito multicêntrico (Multi-NBCAL) conduzido entre novembro de 2018 e novembro de 2019 em seis cidades de diferentes regiões brasileiras. Em 26 hospitais públicos e privados foram entrevistados pediatras, nutricionistas, fonoaudiólogos e um membro da chefia, mediante questionário estruturado. Foram realizadas análises descritivas do conhecimento dos profissionais de saúde sobre a Norma Brasileira de Comercialização de Alimentos para Lactentes e Crianças de Primeira Infância, Bicos, Chupetas e Mamadeiras (NBCAL), das empresas patrocinadoras de eventos científicos e dos patrocínios financeiros ou materiais recebidos, conforme a categoria profissional.

**RESULTADOS:**

Foram entrevistados 217 profissionais de saúde, principalmente pediatras (48,8%). Pouco mais da metade dos profissionais (54,4%) afirmaram conhecer a NBCAL, principalmente em Hospitais Amigos da Criança. A maior parte (85,7%) dos profissionais de saúde havia participado de congressos científicos nos últimos dois anos, mais da metade, 54,3%, deles apoiados pela indústria de substitutos do leite materno, em especial pela Nestlé (85,1%) e Danone (65,3%). Patrocínios foram recebidos por esses profissionais nos eventos, como materiais de escritório (49,5%), refeições ou convites para festas (29,9%), brindes (21,6%), pagamento de inscrição (6,2%) ou de passagem para o congresso (2,1%).

**CONCLUSÃO:**

As indústrias de alimentos infantis infringem a NBCAL ao assediar profissionais de saúde em congressos científicos, oferecendo patrocínios materiais e financeiros diversos.

## INTRODUÇÃO

Os primeiros anos de vida de uma criança são caracterizados pela velocidade de crescimento e desenvolvimento, sendo o leite materno reconhecido como padrão ouro para a nutrição infantil, pois apresenta função moduladora ^1^ , reduz a morbimortalidade infantil e confere proteção imunológica à criança ^2^ . A Organização Mundial de Saúde (OMS) o recomenda de forma exclusiva durante os seis primeiros meses de vida e complementado por alimentos saudáveis até os dois anos de idade ou mais ^3^ .

Visando à proteção da amamentação, a OMS e o Fundo das Nações Unidas para a Infância (Unicef), em Reunião Conjunta sobre Alimentação do Lactente e de Crianças Pequenas, em 1979, propuseram a criação de um conjunto de normas regulatórias que deram origem, em 1981, ao Código Internacional dos Substitutos do Leite Materno ^4^ .

Inspirado no Código, foi criada em 1988, no Brasil, a primeira legislação de regulação da propaganda e comercialização de substitutos do leite materno, que sofreu várias atualizações ao longo dos anos, passando a ser denominada Norma Brasileira de Comercialização de Alimentos para Lactentes e Crianças de Primeira Infância, Bicos, Chupetas e Mamadeiras (NBCAL) e adquirindo força de Lei em 2006 ^5^ . A Lei 11.265/2006 estabelece que fabricantes de fórmulas infantis, fórmulas para crianças de primeira infância, leites, alimentos de transição e produtos de puericultura correlatos e distribuidores desses produtos não podem conceder patrocínios financeiros ou materiais a pessoas físicas, sendo tal patrocínio permitido apenas a entidades científicas de ensino e pesquisa ou a entidades associativas de pediatras e nutricionistas reconhecidas nacionalmente ^6^ . O Decreto 9.579/2018, que regulamenta essa Lei, define patrocínio como o custeio de materiais, pesquisas, evento ou o custeio de profissionais de saúde para participação em atividades ou incentivo de qualquer espécie. É concedido às empresas o fornecimento de uma amostra de fórmula ou alimento infantil a pediatras ou nutricionistas, por ocasião do lançamento do produto ^7^ . A Agência Nacional de Vigilância Sanitária tem a responsabilidade de supervisionar e penalizar as empresas que infringem a NBCAL ^8^ .

Embora o governo e organizações da sociedade civil tenham investido na proteção à amamentação, as estratégias de marketing das corporações multinacionais fabricantes de substitutos do leite materno se renovam continuamente e têm sido efetivas no aumento das vendas, o que mostra a importância de leis nacionais abrangentes e da fiscalização adequada das suas práticas ^9^ . As companhias produtoras de fórmulas infantis, bicos, chupetas e mamadeiras têm mostrado seus produtos como tão bons ou superiores ao aleitamento materno ^9^ e se utilizado da influência e ascendência dos profissionais de saúde sobre as mães e seus familiares para aumentar seu mercado, como estratégias de marketing ^10^ .

A indústria de alimentos infantis participa de eventos científicos para apresentar seus produtos e influenciar profissionais de saúde. O assédio da indústria tem como finalidade criar vínculos com profissionais, professores e estudantes da área da saúde ^11^ . O presente estudo teve por objetivo analisar o recebimento de patrocínios da indústria de substitutos do leite materno por profissionais de saúde em eventos científicos.

## MÉTODOS

Trata-se de estudo transversal, vertente do Estudo Multicêntrico de Avaliação do Cumprimento da Norma Brasileira de Comercialização de Alimentos para Lactentes e Crianças de Primeira Infância, Bicos, Chupetas e Mamadeiras (Multi-NBCAL). Este projeto foi submetido ao comitê de ética em pesquisa da Fiocruz e aprovado pelo Parecer n ^o^ 2.912.729/2018.

A equipe de pesquisa foi composta por coordenador e vice-coordenador com expertise em inquéritos e na NBCAL, bem como um supervisor de campo e um a quatro entrevistadores por cidade. Todos os supervisores e entrevistadores foram capacitados em cursos teórico-práticos de 20 horas sobre a NBCAL, que compreenderam sessões práticas de avaliação do seu cumprimento em hospitais, farmácias e supermercados e foram instrumentalizados em coleta de dados online.

O presente estudo, realizado em hospitais que possuíam leitos obstétricos, seguiu protocolo adaptado do *Netcode Periodic Assessment* ( *Netcode* ) ^12^ , que preconiza a seleção aleatória de 10 maternidades na capital ou na maior cidade do país para avaliação, assim como a seleção de mais maternidades, em outros cenários, caso os recursos financeiros o permitam.

A coordenação da pesquisa optou por comparar hospitais de diferentes tipos, situados em capitais com Índíces de Desenvolvimento Humano Municipal diferentes, bem como em uma cidade do interior brasileiro, e não concentrar a pesquisa apenas na capital ou maior cidade, devido à grande diversidade e extensão de nosso país. As cidades foram escolhidas intencionalmente, segundo a disponibilidade de instituições e pesquisadores dedicados à temática ^13^ . A pesquisa foi conduzida entre novembro de 2018 e novembro de 2019 em seis cidades de diferentes regiões do Brasil: Rio de Janeiro/RJ (seis hospitais), São Paulo/SP (cinco hospitais), Ouro Preto/MG (um hospital), Florianópolis/SC (quatro hospitais), João Pessoa/PB (seis hospitais) e Brasília/DF (quatro hospitais).

Em cada cidade foram listados todos os hospitais com leitos obstétricos com mais de 500 partos por ano, por meio de consulta ao Cadastro Nacional de Estabelecimentos de Saúde (CNES) ^a^ . Os hospitais foram estratificados em hospitais públicos credenciados na Iniciativa Hospital Amigo da Criança (IHAC), hospitais públicos não credenciados na IHAC e hospitais privados. A pesquisa não contemplou Hospitais Amigos da Criança da rede privada, pois, à época, o Brasil só dispunha de nove hospitais dessa categoria, todos situados em cidades do interior ^b^ . A amostra visava contemplar dois hospitais por estrato em cada cidade, a serem selecionados ao acaso se houvesse um número superior a essa quantidade no município. Porém, Florianópolis/SC só dispunha de quatro hospitais com leitos obstétricos, sendo todos os hospitais públicos Amigos da Criança. Ouro Preto/MG só dispunha de um hospital com leitos obstétricos, também Amigo da Criança. Em João Pessoa todos os hospitais públicos eram credenciados nessa iniciativa. Em duas cidades, três maternidades se recusaram a participar do estudo e dificuldades para a obtenção dos termos de aceite institucional foram barreiras que impediram o alcance da amostra previamente estabelecida ^13^ . Foram avaliados, ao final do estudo, 11 hospitais amigos da criança, seis públicos não credenciados e nove privados.

Os entrevistadores foram orientados a realizar a pesquisa em único dia, entrevistando presencialmente em cada maternidade um membro da chefia, cinco pediatras e cinco nutricionistas e/ou fonoaudiólogos, um número fixo de profissionais por maternidade, segundo o protocolo de monitoramento do Netcode ^12^ . Se na maternidade estivessem trabalhando menos de dez profissionais dessas categorias no dia da pesquisa, todos eram entrevistados. Se mais de dez profissionais estivessem atuando, era solicitada à chefia a lista de pediatras, nutricionistas e fonoaudiólogos presentes no dia da entrevista, sendo selecionados, de forma aleatória, até cinco pediatras, até cinco nutricionistas e/ou fonoaudiólogos e uma chefia por maternidade. Todas as entrevistas foram conduzidas mediante assinatura de termo de consentimento livre e esclarecido ^13^ .

Para a realização das entrevistas foi utilizado um formulário eletrônico online por meio do aplicativo MAGPI, instalado em celulares com sistema operacional Android, com interface intuitiva para a entrada de dados, hospedagem eletrônica em servidores da Fiocruz, acompanhamento online da coleta de dados e possibilidade de exportação de dados para os pacotes estatísticos mais comuns ^14^ .

O questionário empregado foi adaptado do questionário de monitoramento da Rede Internacional em Defesa do Direito de Amamentar (em inglês: *International Baby Food Action Network* – IBFAN) ^c^ e do Netcode ^12^ , com validação de conteúdo realizada por painel de especialistas ^13^ . Os entrevistados foram questionados presencialmente sobre: (1) perfil do profissional de saúde: sexo, faixa etária, cor da pele, profissão, tempo de formado e setores de atuação do profissional de saúde (podendo ser citado mais de um setor: alojamento conjunto, unidade neonatal, banco de leite humano); (2) conhecimento acerca da NBCAL (sim, regular – que correspondeu às respostas tenho noção/já ouvi falar, não); citou pelo menos um produto abrangido pela NBCAL (sim – compreendendo fórmula infantil, bico, mamadeira, chupeta, fórmula para criança de primeira infância, leite, ou alimento de transição; não); capacitação na NBCAL por meio de curso ou aula (sim, não); órgão responsável pela capacitação (órgão governamental, conselho profissional, indústria, particular – que correspondeu a: universidade particular/hospital particular/curso pago, outra empresa como distribuidores); capacitação em alimentação infantil (sim, não); (3) participação em congresso ou simpósio sobre alimentação infantil nos últimos dois anos (sim, não), apoio do evento por indústria de alimentos infantis ou produtos correlatos (sim, não), empresa patrocinadora (podendo ser citada mais de uma empresa: Nestlé, Danone, Abbott, Mead Johnson, outra), recebimento de patrocínio pelo profissional de saúde (podendo ser citado mais de um: pagamento de inscrição no congresso, pagamento de passagem, refeição ou festa durante o evento, material de escritório, brinde), recebimento de amostra grátis de substituto de leite materno (sim, não).

Foram realizadas análises descritivas da distribuição dos profissionais de saúde entrevistados por cidade, da caracterização do seu perfil, de seu conhecimento sobre a NBCAL, sua capacitação na NBCAL, o órgão responsável pelo curso e sua capacitação em alimentação infantil, segundo o tipo de hospital. Foi calculada a proporção de cada órgão responsável pela capacitação na NBCAL apenas entre os profissionais capacitados na mesma.

Para verificar a homogeneidade das características dos profissionais de saúde, assim como seu conhecimento e capacitação na NBCAL e em alimentação infantil, segundo o tipo de hospital, foi aplicado o teste qui-quadrado de Pearson, considerando o nível de significância estatística de 5%.

Foi analisada a proporção de profissionais de saúde que havia participado de eventos científicos nos dois últimos anos, segundo categoria profissional. Dentre os que relataram participação em eventos científicos, foi calculada a proporção dos que referiram que o evento foi apoiado por indústria de substitutos do leite materno, sendo descritas as empresas que patrocinaram esses eventos, cada profissional podendo citar mais de uma empresa.

Por fim, dentre os profissionais de saúde que participaram de eventos científicos apoiados pela indústria, foi analisada a frequência dos que receberam patrocínios materiais ou financeiros nesses eventos, bem como a frequência dos que receberam amostras grátis de substitutos do leite materno, segundo a categoria profissional.

## RESULTADOS

Foram entrevistados ao todo 217 profissionais de saúde que atuavam em 26 hospitais de seis cidades brasileiras. Desses profissionais, 44,2% trabalhavam em hospitais públicos credenciados na IHAC; 26,3% em hospitais públicos não credenciados na IHAC e 29,5% em hospitais privados. Rio de Janeiro, São Paulo e João Pessoa foram as cidades com mais profissionais de saúde entrevistados ( Tabela 1 ). Foram registradas quatro recusas entre os profissionais de saúde abordados (1,8%).

**Tabela 1:** Profissionais de saúde entrevistados por município, segundo o tipo de hospital. Multi-NBCAL, 2018–2019.

Município	Públicos Amigos da Criança		Públicos não credenciados		Privados		Total
	n	%	n	%	n	%	n
Rio de Janeiro	25	37,3	31	46,3	11	16,4	67
São Paulo	9	22,5	19	47,5	12	30	40
Ouro Preto	6	100	0	0	0	0	6
Florianópolis	18	56,3	0	0	14	43,8	32
João Pessoa	30	66,7	0	0	15	33,3	45
Brasília	8	29,6	7	25,9	12	44,4	27
Total	96	44,2	57	26,3	64	29,5	217

NBCAL: Norma Brasileira de Comercialização de Alimentos para Lactentes e Crianças de Primeira Infância, Bicos, Chupetas e Mamadeiras.

A maioria dos profissionais entrevistados era do sexo feminino, principalmente na rede privada. Houve um predomínio de profissionais de saúde de cor branca, exceto na rede particular, e 12,4% dos profissionais não declararam a sua cor. Entre os entrevistados, 48,8% eram pediatras, 29% nutricionistas, 18% fonoaudiólogos e 4,1% de outras profissões em posição de chefia, e essa distribuição foi relativamente homogênea entre os diferentes tipos de hospitais. A maioria dos profissionais tinha entre 35 e 59 anos de idade e entre 11 e 24 anos como profissional formado. Os setores de atuação mais frequentes foram unidade neonatal e alojamento conjunto ( Tabela 2 ).

**Tabela 2:** Características dos profissionais de saúde entrevistados, segundo o tipo de hospital. Multi-NBCAL, 2018–2019.

Características	Públicos Amigos da Criança		Públicos não credenciados		Privados		Total		p
	n	%	n	%	n	%	n	%	
Sexo									
Masculino	17	17,7	8	14	2	3,1	27	12,4	0,022
Feminino	79	82,3	49	86	62	96,9	190	87,6	
Idade									
20–34	24	25	13	22,8	24	37,5	61	28,1	0,247
35–59	65	67,7	39	68,4	33	51,6	137	63,1	
≥ 60	7	7,3	5	8,8	7	10,9	19	8,8	
Cor									
Branca	63	65,6	37	64,9	25	39,1	125	57,6	0,001
Preta	5	5,2	2	3,5	9	14,1	16	7,4	
Parda	23	24	6	10,5	18	28,1	47	21,7	
Amarela	1	1	1	1,8	0	0	2	0,9	
Não declarada	4	4,2	11	19,3	12	18,8	27	12,4	
Profissão									
Pediatra	45	46,9	32	56,1	29	45,3	106	48,8	0,184
Nutricionista	25	26	19	33,3	19	29,7	63	29	
Fonoaudiólogo	19	19,8	6	10,5	14	21,9	39	18	
Outra	7	7,3	0	0	2	3,1	9	4,1	
Tempo de formado									
≤ 10 anos	29	30,2	17	29,8	27	42,2	73	33,6	0,250
11–24 anos	41	42,7	19	33,3	23	35,9	83	38,2	
≥ 25 anos	26	27,1	21	36,8	14	21,9	61	28,1	
Setores de atuação									
Alojamento conjunto	50	52,1	29	50,9	32	50	111	51,2	0,966
UTI neonatal	57	59,4	34	59,6	46	71,9	137	63,1	0,225
Banco de leite humano	7	7,3	6	10,5	5	7,8	18	8,3	0,771
Total	96	100	57	100	64	100	217	100	

NBCAL: Norma Brasileira de Comercialização de Alimentos para Lactentes e Crianças de Primeira Infância, Bicos, Chupetas e Mamadeiras; UTI: unidade de terapia intensiva.

Relataram conhecer a NBCAL 54,4% dos profissionais de saúde e 59,9% souberam citar pelo menos um produto abrangido por essa legislação. Esse conhecimento foi superior em profissionais que atuavam em Hospitais Amigos da Criança e inferior naqueles lotados em hospitais públicos não credenciados. A participação em curso ou aula sobre a NBCAL foi de 30%, maior entre profissionais que atuavam em Hospitais Amigos da Criança (40,6%). Entre os capacitados na NBCAL, 93,8% relataram que o mesmo foi realizado por órgão governamental. A participação em curso ou aula sobre alimentação infantil foi alta (93,5%) em todos os tipos de hospitais ( Tabela 3 ).

**Tabela 3:** Conhecimento dos profissionais de saúde sobre a NBCAL, capacitação na NBCAL e em alimentação infantil, segundo o tipo de hospital. Multi-NBCAL, 2018–2019.

Conhecimento/capacitação	Públicos Amigos da Criança		Públicos não credenciados		Privados		Total		p
	n	%	n	%	n	%	n	%	
Conhece NBCAL									
Sim	65	67,7	23	40,4	30	46,9	118	54,4	0,001
Regular ^a^	14	14,6	6	10,5	13	20,3	33	15,2	
Não	17	17,7	28	49,1	21	32,8	66	30,4	
Citou produtos NBCAL									
Sim	73	76	25	43,9	32	50	130	59,9	< 0,001
Não	23	24	32	56,1	32	50	87	40,1	
Curso/aula sobre NBCAL									
Sim	39	40,6	14	24,6	12	18,8	65	30	0,007
Não	57	59,4	43	75,4	52	81,3	152	70	
Responsável pelo curso									
Órgãos do governo	39	100	13	92,9	9	75	61	93,8	0,001
Conselhos	0	0	0	0	0	0	0	0	
Indústria	0	0	0	0	0	0	0	0	
Particular ^b^	0	0	1	7,1	2	16,7	3	4,6	0,243
Outro	0	0	0	0	1	8,3	1	1,5	0,301
Curso/aula sobre alimentação infantil									
Sim	92	95,8	50	87,7	61	95,3	203	93,5	0,113
Não	4	4,2	7	12,3	3	4,7	14	6,5	
Total	96	100	57	100	64	100	217	100	

NBCAL: Norma Brasileira de Comercialização de Alimentos para Lactentes e Crianças de Primeira Infância, Bicos, Chupetas e Mamadeiras.

^a^ Regular corresponde às respostas: “tenho noção” ou “já ouvi falar”.

^b^ Particular corresponde às respostas: universidade ou hospital particular ou curso pago.

Grande parte dos profissionais de saúde havia participado de congresso ou simpósio científico nos dois últimos anos (85,7%) e 54,3% deles relataram que empresas de substitutos do leite materno apoiaram esses eventos, principalmente pediatras (71,1%). Nestlé (85,1%) e Danone (65,3%) foram as empresas mais citadas como patrocinadoras de eventos científicos ( Tabela 4 ).

**Tabela 4:** Participação em eventos científicos, patrocínio da indústria a esses eventos e empresas patrocinadoras, segundo categoria profissional. Multi-NBCAL, 2018–2019.

Participação/apoio	Pediatra		Nutricionista		Fonoaudiólogo		Outros		Total	
	n	%	n	%	n	%	n	%	n	%
Participação em congresso										
Sim	97	91,5	45	71,4	37	94,9	7	77,8	186	85,7
Não	9	8,5	18	28,6	2	5,1	2	22,2	31	14,3
Evento apoiado pela indústria ^a^										
Sim	69	71,1	18	40	13	35,1	1	14,3	101	54,3
Não	28	28,9	27	60	24	64,9	6	85,7	85	45,7
Empresa patrocinadora ^b^										
Nestlé	60	87	14	77,8	11	84,6	1	100	86	85,1
Danone	50	72,5	9	50	6	46,2	1	100	66	65,3
Abbott	27	39,1	3	16,7	0	0	1	100	31	30,7
Mead Johnson	27	39,1	3	16,7	0	0	0	0	30	29,7
Outra	3	4,3	2	11,1	0	0	0	0	5	5

NBCAL: Norma Brasileira de Comercialização de Alimentos para Lactentes e Crianças de Primeira Infância, Bicos, Chupetas e Mamadeiras.

^a^ Calculado entre os profissionais que participaram de congresso nos últimos dois anos.

^b^ Cada profissional poderia citar mais de uma empresa patrocinadora.

A maior parte dos profissionais de saúde que participaram de eventos científicos apoiados pela indústria relataram ter recebido patrocínio financeiro ou material da indústria de substitutos do leite materno nesses eventos, como materiais de escritório (49,5%), refeições ou festas no evento (29,9%), brindes (21,6%), pagamento de inscrição (6,2%) ou de passagem para o congresso (2,1%), cerca de um terço dos entrevistados afirmaram não terem recebido patrocínio. Receberam amostras grátis de substitutos do leite materno 22,7% dos profissionais de saúde: pediatras (25%), nutricionistas (18,8%) e também um fonoaudiógo e outro profissional de saúde ( Tabela 5 ).

**Tabela 5:** Recebimento de patrocínios e de amostras grátis em congressos científicos, segundo categoria profissional. Multi-NBCAL, 2018–2019.

Patrocínio	Pediatra		Nutricionista		Fonoaudiólogo		Outros		Total	
	n	% ^a^	n	%	n	%	n	%	n	%
Patrocínio financeiro ou material no congresso										
Material de escritório	30	44,1	10	62,5	8	66,7	0	0	48	49,5
Refeição ou festa	21	30,9	6	37,5	2	16,7	0	0	29	29,9
Brinde	15	22,1	5	31,2	1	8,3	0	0	21	21,6
Inscrição no congresso	3	4,4	2	12,5	1	8,3	0	0	6	6,2
Passagem	2	2,9	0	0	0	0	0	0	2	2,1
Não recebeu incentivo	27	39,7	2	12,5	4	33,3	1	100	34	35,1
Amostra no congresso										
Amostra grátis	17	25	3	18,8	1	8,3	1	100	22	22,7
Total	68	100	16	100	12	100	1	100	97	100

NBCAL: Norma Brasileira de Comercialização de Alimentos para Lactentes e Crianças de Primeira Infância, Bicos, Chupetas e Mamadeiras.

^a^ Percentuais dessa tabela calculados a partir do total de profissionais que participaram de eventos apoiados pela indústria (houve quatro não respostas).

## DISCUSSÃO

Apesar de vedado pela Lei 11.265/2006, quase dois terços dos profissionais que participaram de eventos científicos apoiados pela indústria referiram o recebimento de patrocínio financeiro ou material nesses eventos. Pediatras, nutricionistas e fonoaudiólogos foram assediados pela indústria de substitutos de leite materno, tanto pelo pagamento de inscrições e passagens para os congressos, itens de custo mais alto, como pelo convite a festas ou refeições nos eventos ou mesmo a oferta de sacolas, canetas, blocos, *notepads* , calendários e brindes, itens de mais baixo custo. Além de assediar profissionais de saúde por meio de financiamento e brindes, as indústrias de substitutos do leite materno disseminam informação e conceitos favoráveis ao uso de seus produtos em eventos científicos ^15^ , buscando influenciar pediatras, cuja relação com os pacientes é considerada “fiduciária” na prescrição desses produtos, pois têm conhecimento especializado e experiência e detêm a confiança de todos ^16^ . As indústrias estabelecem também relações com líderes de opinião e com organizações de saúde, buscando influenciar programas de saúde por meio de acesso e colocação de atores em contextos de políticas governamentais ^15^ . Buscam, ainda, reter avanços em medidas regulatórias, impedir a aprovação de legislações, postergar a sua implementação ou fazer retroceder medidas já implementadas ^17^ .

A promoção de substitutos do leite materno por seus fabricantes e distribuidores é uma barreira global para o crescimento da amamentação no mundo ^18 , 19^ , em especial nos países de baixa e média renda, onde as vendas dos substitutos do leite materno crescem em uma progressão de 10% ao ano ^20^ . Nesses países, apenas 37% das crianças menores de seis meses de idade são amamentadas exclusivamente e avanços na prática da amamentação poderiam salvar até 823.000 crianças todos os anos ^2^ .

A NBCAL não permite o patrocínio da indústria de substitutos do leite materno a pessoas físicas, visando proteger as mães de pressões comerciais inadequadas, visto que as companhias produtoras de alimentos infantis se utilizam de várias estratégias para obter a fidelidade das mães ao consumo de seus produtos ^21^ .

Contudo, no presente estudo, cerca de 60% dos pediatras que participaram de eventos científicos apoiados pela indústria receberam patrocínios materiais ou financeiros nesses eventos. Desses, mais de 40% receberam materiais de escritório e quase um terço, convites para festas ou refeições. Nutricionistas e fonoaudiólogos também foram bastante assediados pela indústria de alimentos infantis. Estudo realizado na Indonésia igualmente aferiu oferta a profissionais de saúde de ímãs, pôsteres, termômetros, calendários e gráficos de crescimento, bem como visitas frequentes aos mesmos por representantes de companhias multinacionais de alimentos infantis ^22^ . A aceitação de presentes pelos profissionais de saúde pode se constituir em promoção indireta das empresas produtoras de substitutos do leite materno, em detrimento do apoio à prática da amamentação. O porte de brindes com o logotipo de empresas de alimentos infantis pelos profissionais de saúde pode ser percebido pelos seus pacientes como um endosso ao uso desses produtos ^23^ .

Grummer-Strawn et al. ^24^ , utilizando dados coletados na internet, identificaram uma forte presença da indústria junto à categoria profissional dos pediatras, pois 60% das associações pediátricas em todo o mundo recebiam algum tipo de apoio financeiro da indústria de substitutos do leite materno, principalmente nas Américas (82%), na Europa (66%) e na Ásia (50%). Essa realidade se repete no Brasil, onde parceria entre a Sociedade Brasileira de Pediatria e uma indústria de substitutos de leite materno resultou no “Programa Jovens Pediatras: Nestlé Nutrition”, uma plataforma dirigida a médicos residentes de pediatria para influenciar a sua formação profissional ^d^ .

O patrocínio da indústria a um congresso de saúde ou nutrição infantil funciona como um endosso do congresso àquela marca. O recebimento de patrocínio coloca a entidade promotora em dívida com a indústria de substitutos do leite materno e referenda o uso de seus produtos ou práticas ^10^ . O patrocínio por parte de fabricantes de substitutos do leite materno, assim como a oferta de apoio financeiro por parte dessas empresas, pode criar fidelidade do profissional de saúde e das associações profissionais às empresas de alimentos infantis ^22^ . Rea e Toma ^25^ , num dos primeiros estudos nacionais sobre o tema, ressaltam os conflitos de interesse envolvidos na relação profissional de saúde – indústria, apresentando resultados de um monitoramento da NBCAL realizado em mais de 30 cidades brasileiras, que observou formas de aliciamento de profissionais de saúde pela indústria de fórmulas infantis, como a concessão de passagens aéreas, busca do profissional no aeroporto e inscrição em congresso.

Entre as companhias multinacionais de fórmulas infantis, a Nestlé se destacou como a mais citada pelos profissionais de saúde no patrocínio de eventos científicos. O Brasil é o segundo maior mercado e o quinto maior país em faturamento da Nestlé, fazendo dela a líder no setor alimentício nacional, com 15 filiais espalhadas por todo o território brasileiro, além de cinco centros de distribuição, quatro depósitos e 26 unidades fabris ^26^ . Do mesmo modo, no México, a Nestlé foi indicada por gestores entrevistados em hospitais públicos e particulares como a principal fabricante de alimentos infantis a entrar em contato com eles ^27^ .

Apesar de mais da metade dos profissionais de saúde ter afirmado conhecer a NBCAL e quase 60% terem sido capazes de citar ao menos um produto abrangido pela Norma, menos de um terço tinha frequentado algum curso ou aula sobre a temática. A maior parte dos cursos ou aulas sobre a NBCAL foi ministrada por órgãos governamentais. A ausência de capacitações promovidas por conselhos ou associações profissionais chama a atenção, pois é necessário um maior empenho dessas entidades na formação de profissionais para o cumprimento da NBCAL e do Código Internacional de Substitutos do Leite Materno. Uma maior familiaridade dos profissionais de saúde com essa importante legislação de proteção à amamentação não impediria o aliciamento de parte desses profissionais pela indústria de substitutos de leite materno, mas poderia aumentar sua percepção sobre os conflitos de interesse envolvidos nessa relação e reduzir as violações a essa Lei, contribuindo para a promoção à saúde da comunidade ^28^ .

Atualmente os currículos das escolas de medicina estão se dedicando mais a ensinar seus alunos a alimentar bebês com fórmula infantil do que em apoiar as mães a amamentar usando habilidades de aconselhamento ^29^ . Estudo conduzido com discentes da Faculdade de Medicina da Universidade Federal de Alagoas verificou um baixo conhecimento de alunos do último período sobre o Código Internacional e sobre a NBCAL, mostrando que há muito a ser feito para promover o ensino acerca do aleitamento materno no curso médico ^30^ .

No presente estudo, o conhecimento e capacitação na NBCAL foram mais frequentes em profissionais atuando em Hospitais Amigos da Criança, no entanto era esperado que sua totalidade conhecesse essa legislação, conteúdo obrigatório na sua formação. É importante reforçar a necessidade da educação continuada, para que profissionais capacitados há mais tempo possam aprimorar seus conhecimentos e profissionais admitidos após o credenciamento desses hospitais na IHAC sejam sistematicamente capacitados ^31^ . A IHAC foi criada para fornecer maior apoio à prática da amamentação nos hospitais com leitos obstétricos, por meio da capacitação da equipe de saúde e da adequação das rotinas hospitalares, sendo o cumprimento do Código Internacional de Substitutos do Leite Materno um dos critérios a serem preenchidos para a adesão ao primeiro dos Dez Passos para o Sucesso do Aleitamento Materno ^24^ , iniciativa considerada como uma importante estratégia, com impacto positivo nas taxas de aleitamento materno e inserida na agenda de desenvolvimento sustentável da Organização das Nações Unidas ^31 , 32^ . A sustentabilidade de suas ações vem sendo alvo de monitoramento contínuo no Brasil ^33^ .

Limitações da presente pesquisa devem ser apontadas: não se sabe se as amostras recebidas pelos pediatras e nutricionistas entrevistados correspondiam a produtos sendo lançados, portanto, não se pode avaliar em que extensão a indústria estava descumprindo a Lei 11.265/2006 nesse aspecto, mas no presente estudo outros profissionais de saúde também receberam amostras de substitutos do leite materno, o que contraria a NBCAL ^7^ . Outra limitação a ser ressaltada é que os profissionais foram perguntados sobre informações relativas ao recebimento de patrocínios em congressos nos dois últimos anos, sujeitas a viés de memória, uma vez que deve ser mais recorrente o esquecimento do recebimento de patrocínios do que o relato de recebimento de patrocínios não existentes. Esse viés pode ter levado à subestimação dos patrocínios recebidos, especialmente de brindes de menor valor.

A metodologia proposta pelo Netcode ^12^ se propõe a identificar os tipos mais recorrentes de infrações ao Código Internacional ^4^ (e à NBCAL ^6^ ) pelo seu método de seleção aleatória de maternidades e entrevistas, a Multi-NBCAL adaptou esse protocolo, avaliando 26 maternidades de diferentes cidades, entre elas a capital e a maior cidade do país. A limitação desta pesquisa está em sua amostra não ser probabilística, o que reduz a possibilidade de generalização dos resultados, o que significa que os achados do presente estudo refletem a realidade dos profissionais entrevistados nas maternidades incluídas, não devendo ser interpretados como a realidade das cidades avaliadas. Contudo, os resultados deste estudo multicêntrico são suficientes para promover o debate sobre potenciais conflitos de interesse das práticas profissionais ante o assédio das indústrias de produtos que competem com o aleitamento materno.

Um estudo de confiabilidade do questionário se faz necessário para avaliação das suas propriedades psicométricas, contudo, a supervisão constante da aplicação da grande maioria dos questionários por pesquisadores experientes, aliado ao rigoroso treinamento dos entrevistadores, pode ter reduzido uma potencial variabilidade interobservadores. Uma análise de confiabilidade do questionário encontrou um Alfa de Cronbach de 0,794 para o bloco de perguntas avaliadas no presente estudo (dados não tabulados), padrão considerado elevado.

Conclui-se que estratégias de marketing da indústria dirigidas a profissionais de saúde são largamente praticadas em espaços de formação, como congressos e simpósios científicos, indicando que a existência de uma regulamentação, mesmo na forma de Lei, não é suficiente para coibir práticas ilegais de promoção de substitutos do leite materno, apesar do seu impacto sobre a saúde infantil ser amplamente conhecido ^34^ . A inadequada fiscalização de eventos científicos permite que companhias multinacionais de substitutos do leite materno busquem indevidamente envolver profissionais de saúde no marketing dos seus produtos sem qualquer punição ^19^ .

Recomenda-se o desenvolvimento de ações governamentais que visem aumentar o conhecimento e a adesão à legislação vigente, tanto pelas empresas quanto pelos profissionais de saúde, e que a temática dos conflitos de interesse seja abordada desde os cursos de graduação da área da saúde. Como nenhum profissional de saúde tem mais ascendência sobre as mães do que os pediatras, que não só recomendam, como também prescrevem fórmulas infantis, as associações de pediatria devem colaborar com a formação continuada de seus profissionais dentro de um compromisso ético com o aleitamento materno e a saúde infantil. Também é necessário que a vigilância sanitária tenha uma atuação efetiva na fiscalização da NBCAL para um melhor cumprimento da legislação vigente. É urgente que situações de conflito de interesses entre a indústria e os profissionais de saúde sejam prevenidas e coibidas, objetivando a proteção ao aleitamento materno e à saúde infantil.

## Footnotes

a Ministério da Saúde (BR), DATASUS. Cadastro Nacional de Estabelecimentos de Saúde – CNES. Brasília, DF; s.d. [citado 22 mai 2022]. Disponível em: https://cnes.datasus.gov.br/

b Ministério da Saúde. Portal Saúde da Criança NET. IHAC - Iniciativa Hospital Amigo da Criança [citado 22 mai 2022]. Disponível em: http://sisac.datasus.gov.br/saudedacrianca/ihacSobre.html#

c Rede Internacional em Defesa do Direito de Alimentar. Belo Horizonte, MG: IBFAN Brasil; 2022 [citado 22 mai 2022]. Disponível em: http://www.ibfan.org.br

d Aliança pela Alimentação Adequada e Saudável. Porto Alegre, RS; c2020 [citado 10 jul 2020]. Disponível em: https://alimentacaosaudavel.org.br/blog/organizacoes-denunciam-conflito-de-interesse-em-curso-da-nestle-para-medicos-residentes-de-pediatria/8180/
